# A conceptual framework for reopening our society during the Covid-19 pandemic

**DOI:** 10.12688/f1000research.24352.1

**Published:** 2020-06-08

**Authors:** Christopher J. Gill, Sandro Galea

**Affiliations:** 1 Department of Global Health, Boston University School of Public Health, Boston, Massachusetts, 02118, USA; 2 School of Public Health, Boston University School of Public Health, Boston, Massachusetts, 02118, USA

**Keywords:** Risk index, Covid 19, CV19, decision making

## Abstract

Decisions about how to go about the necessary task of re-opening our society in the midst of the Covid-19 (CV19) have been paralyzed by our extremes. But we can neither afford to insist on a zero-risk response, nor can we pretend that the risk does not exist. What is needed are tools to rationally triage the risk. To this end, we propose a novel ‘risk index’, which is the intersection of two components of risk: 1) the risk of an individual becoming infected due to action ‘X’; and 2) the likely probability of death (or serious harm) if that individual develops CV19. The risk index allows risk to be compared across different scenarios, and may reveal that seemingly very different situations constitute similar degrees of risk. With risk measured in this way, one can then contrast different levels of risk against the social benefits of absorbing that risk, allowing actions to be sorted into those that are tolerable, debatable, or acceptable. While these concepts are presented in abstract based on approximate estimates of risk and influenced by our judgements about social desirability, the concept itself can be refined as more accurate approximations of risk and broadly accepted values of social desirability are derived empirically. In short, this is a tool intended to provide a useful empirical framework for rationale decision making about CV19.

## Accepting risk is rationale and unavoidable

The current Covid-19 (CV19) public discussion has taken on absolutist shades, ranging between arguments in favor of ever longer massive national shutdowns to arguments in favor of a return to pre-CV19 interactions. These arguments are ultimately arguments about risk tolerance but are seldom framed that way. We would argue that a reasoned approach to risk and our tolerance of risk can help us sort through the options available and to chart a path forward.

Consider, by way of illustration, a recent example. In the third week of May, a music concert was hosted by the Tupelo Music Hall in Derry New Hampshire ^1^. At first glance, this seemed to be a dangerous proposition. But the details of the event were reassuring: It was to be held outdoors at a rented drive-in movie theater. Guests would be admitted after presenting their email confirmation at the gates, guests would park and watch the show from their cars or, if they preferred, while sitting on their own lawn chairs set up next to their cars. And other than going to the bathroom (ventilated portable toilets), social spacing was enforced. Food and drink could be ordered online in advance in the show but was delivered by concert staff who were wearing face masks.

So, while it is true that hosting the event incurred more risk than not hosting the event, in this instance, the organizers had been deliberate, thoughtful, and cautious about their risk mitigation strategies, and were aligned with guidelines issued by the State of New Hampshire ^2^. Overall their plan seemed reasonable.

What the plan highlights is a tolerance of risk. Certainly, all concert goers would have faced less risk had they stayed home. But they, and the state, judged that risk to be acceptable, and the concert went on. Far from being an outlandish exception, this example illustrates an important general point: we accept risks all the time. That is why it is legal to drive a motorcycle, to skydive, to smoke, to drink soda, to buy sailboats, to kiss a stranger in a night club, or to own a pet tarantula. And we make finely tuned decisions about these risks. While it is legal to drive a motorcycle, even though for every mile driven motorcycle drivers have a 35-fold greater risk than car drivers, it is illegal to drive a motorcycle without a helmet in most states (though ironically not in New Hampshire) ^3^. Everything we do involves parsing risk, deciding what is acceptable to us based on other values (e.g., the pleasure of driving a motorcycle) and what crosses a line of intolerable risk (e.g., driving a motorcycle while intoxicated). CV19 is a particular kind of risk—one that is shared because the disease is contagious, and one where we confer risk on each other. It is not, however, unique in that respect. Kissing in bars is a good example of a shared risk: it may result in cold sores (Herpes simplex infections; common but not life threatening) or bacterial meningitis ( *Neisseria meningitidis* infections; rare but lethal).

The Tupelo Music Hall concern therefore constituted a pragmatic example of the kind of social experiments that are urgently needed to help us learn how to reopen our society. Such experiments are all variations on the same theme: how do we balance the risk of an action against the value of that action?

But answering that question is impossible unless we first have a better way of measuring risk.

## The proposed risk index

Aiming to help organize our thinking on this, we suggest that we can conceptualize risk from CV19 as having two components. These are:

1) the probability of getting infected by doing action ‘X’; and

2) the likelihood of dying if one becomes infected.

**Figure 1.**
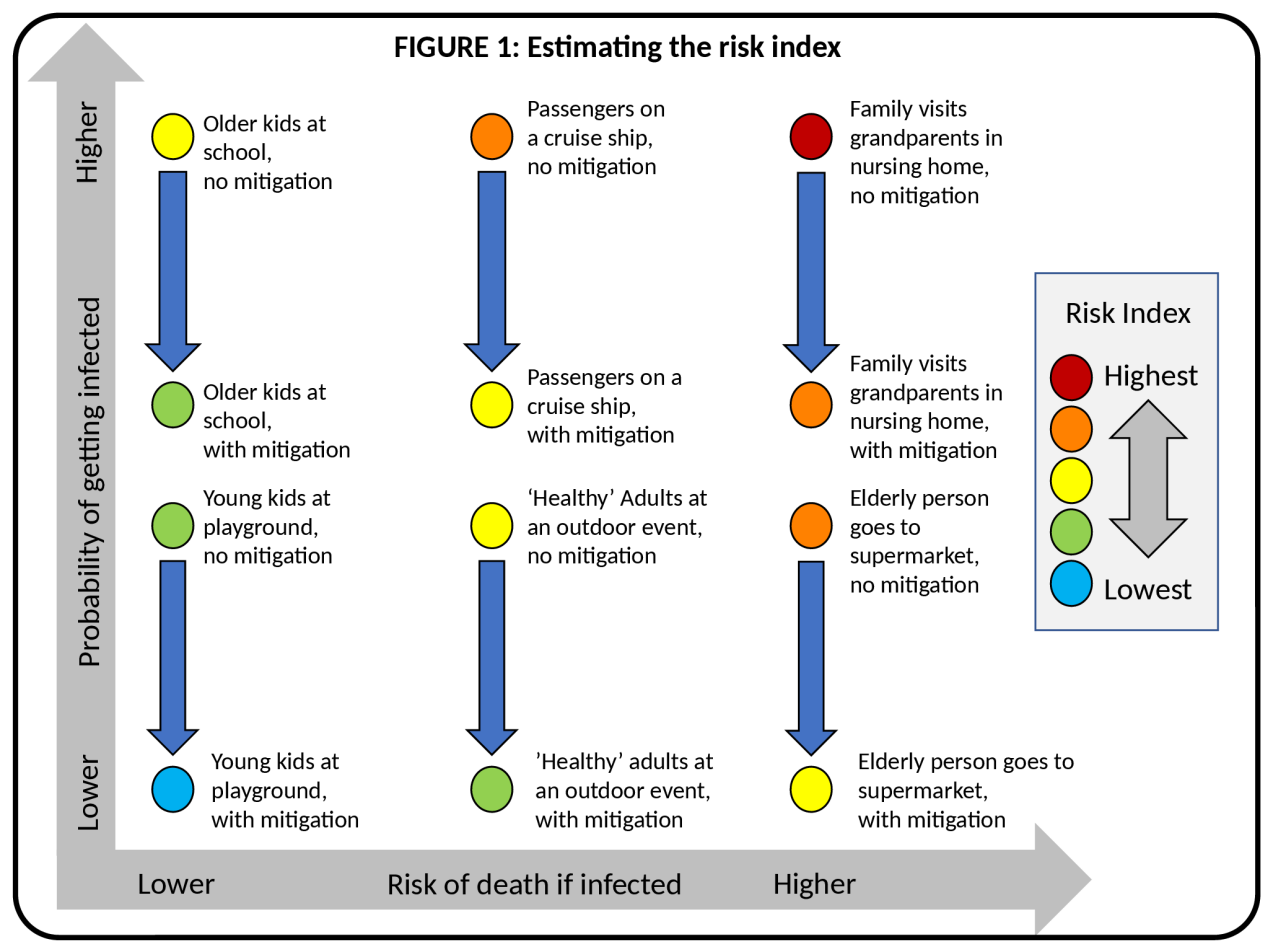
Estimating the risk index.

The intersection of these factors yields a conjoint risk index as shown in Figure 1.

Using best guesses, we could populate the matrix with a set of scenarios illustrating several combinations of risk and vulnerability. For convenience, we color code these combinations from highest to lowest risk index (red>orange>yellow>green>blue). For simplicity, we plot the gradations by primary colors and first order combinations (red and yellow make orange; blue and yellow make green), but obviously the risk index is actually a continuum.

The risk index is bounded by two extreme scenarios. On the bottom left we envision a child at an outdoor playground. Children are rarely (but not never) harmed by CV19, and the risk of transmission when outdoors is low ^4^. With mitigation, this risk could be lower still. For example, kids could wear masks, sanitize hands after play, and we could limit how many play at a given time to reduce crowding.

By contrast, the top right presents the opposite extreme and a worst-case scenario. Here, we can imagine an adult who is unknowingly infected with CV19, who now goes to visit their hypertensive and diabetic grandmother at a nursing home. Indoor transmission is very efficient and the odds are high that the grandmother will not survive an encounter with CV19.

In both cases, the risk index considers the risk solely from the perspective of the individual absorbing the risk (the child or the grandmother). But the framework provides a reminder that we exist in a complicated web of relationships, and that our actions have consequences beyond ourselves. In this way, the framework allows one to separately infer the risk to a hypothetical child or grandmother in isolation, or to model the impact of sequential interactions.

## Advantages and implications of the risk index

This approach has close similarities to the theory underlying Disability Adjusted Life Years (DALYs) ^5^. The DALY is a population level index that measures the impact of any given disease as the combined effect of the total number of years of life lost due to that condition, plus the number of years lived with varying degrees of disability. As with our proposed risk index, it is a way of comparing the net impact of different diseases and can be a valuable tool for public health decision making. Indeed, the DALY is the foundation for the Institute of Health Metrics and Evaluation (IHME) Global Burden of Disease Study ^6^. Other disciplines in public health also index outcomes in different ways.

The key advantage of using a risk index is that it becomes possible for risk to be compared across different individuals and different scenarios. Within the limits of our approximations, the risk index suggests that scenarios that differ markedly in their details, may actually present similar levels of risk. This makes the concept of risk fungible. For example, by taking some best guesses, this model suggests that sending teens back to high school is roughly the same as an elderly person going to the supermarket while wearing a mask.

A further advantage is that the risk index allows us to hypothesize about the potential effectiveness of different risk mitigation strategies. For simplicity, we have assumed that the impact of risk mitigation strategies is equivalent across each of our scenarios, but in reality, this is surely not the case. Different scenarios present different opportunities for risk mitigation strategies, and these may be variably effective, which would be represented by expanding or reducing the risk of transmission.

For example, much attention has focused on the value of reopening schools ^7^. Yet the traditional schooling model remains a high transmission proposition. In the base case, schools are characterized by kids clustered in classes, gyms and cafeterias (or just huddled over shared smartphones), and systematically sharing saliva deliberately (by kissing) or inadvertently (via aerosols, droplets, or shared water bottles). But varying degrees of mitigation are certainly possible. Kids can wear masks; schools may alternate in person vs. distance learning week by week; classes taught by elderly/infirm faculty could all go online (to protect the faculty from the students); the semester could be compressed; windows could be open where possible; and so forth. In fact, there are many ways of reducing the risk index while still largely benefitting from the in-person interactions that add value to student education and satisfy the importance of social engagement in the lives of teens.

While it is theoretically possible to reduce the level of harm if infected (i.e., moving to the left on the X axis), in practice this may not be realistic within the time scale of an epidemic. For example, we know that hypertension, obesity and diabetes increase the risk of CV19 death ^8^. But we do not know whether a radical diet and exercise regimen would reduce CV19 fatality rates at an individual level, and accomplishing that at scale and in within the time scale of an epidemic would be immensely challenging. Thus, for practical purposes, the risk mitigation strategies focus only on reducing the risk of transmission (i.e., moving down on the Y axis).

There may be situations for which there are no effective or practical risk mitigation strategies at all. But that too is an acceptable limitation. This is a starting point for risk assessment that is immediately useful when limited to educated guesses, but which becomes more useful and precise when those approximations are replaced by better estimates of actual risk and actual harm. The index is a place to begin discussions and to draw approximate conclusions, not a place to end. It is also an opportunity to identify situations where more precise data from research would be particularly important, making this a helpful tool to guide research.

## Taking the next step: integrating the risk index with the value of different actions

**Figure 2.**
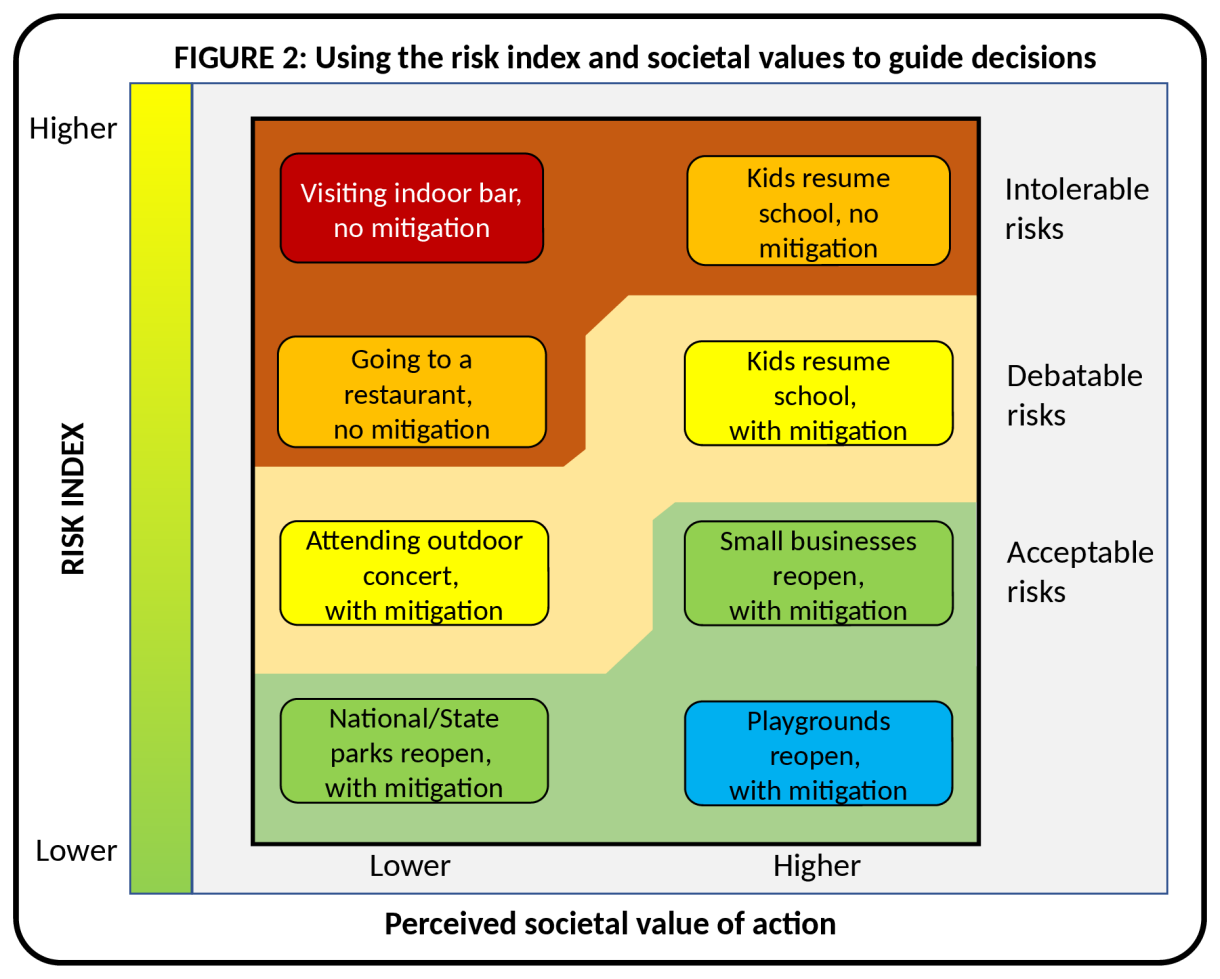
Using the risk index and societal values to guide decisions.

While useful by itself, the risk index is more useful for answering an even more important question: *does the value of a given action justify that risk*? This is shown in Figure 2, where the Y axis is the risk index as estimated from Figure 1, and the new X axis plots the perceived societal value for each action.

In this schema, the easiest decision is to open playgrounds. Kids benefit from freedom, exercise and social interaction and parents need their kids to play and to be happy and to have a break from child care. Moreover, playgrounds exist already and have minimal opening costs. Conversely, opening state or national parks are somewhat more expensive and are not immediately accessible in the same way that playgrounds are, making them of slightly lower value to society, particularly during the lock down. Opening playgrounds is a low risk/high value proposition, colloquially known as a ‘no brainer’. It does not, however, mean that those kids can later visit their grandmother at the nursing home.

While this is certainly debatable, the social value of opening indoor bars seems to us far lower than playgrounds, even though the economic value of bars may be considerable. While their value to society can be argued, from the perspective of the risk index, spending a Friday night in intimate contact with a crowd of strangers is an ideal strategy for spreading the coronavirus. Moreover, it is hard to envision many strategies for risk mitigation that would be acceptable. Patrons cannot drink beer while wearing masks, and masks are a barrier to social intimacy, and thus undermine a key reason why people go to bars. Outdoor bars would be safer, but that puts the drinking out on the street in most cases, which has its own negative consequences. However, one could legitimately reframe this analysis more discretely in terms of economic value (removing elements of moral judgement), social equity, or many other potential permutations of value.

Through these risk index/value combinations we can begin to sort actions into those that are acceptable, debatable, or intolerable. In all likelihood, we will spend a lot of time discussing actions that fall into the debatable range, which is precisely the point. This is what rational decision making looks like.

## Conclusions: Opening society means making thousands of individual risk/value assessments

We fully recognize that this approach leaves many questions unanswered and rests on assumptions about deriving risk index that here are only approximate. However, the goal in proposing this framework is not to categorically rank all different levels of risk or value and thereby answer all potential questions. Rather, the framework is merely a way to start the types of reasoned conversations that must occur if we want to open society in a way that maximizes benefits while minimizing risks.

Currently, our debate on when and how to reopen society has been paralyzed by our absolutist extremes. While it seems certain that the lock down was essential for buying us valuable time to figure out how we respond to the CV19 strategy, this has had devastating consequences to our economy and cannot be sustained indefinitely. But denying the risk of CV19 is also a mistake, which if pursued will result in death on a scale that the US has never seen before (including the 1918 influenza pandemic) and may also result in economic collapse. Neither extreme is tenable.

Rather, we suggest that our way forwards lies somewhere in the rational middle. It means we must accept gradually increasing levels of risk, ideally validated by a rigorous testing strategy to allow us to gauge the impact of these decisions and to shift our strategies if our assumptions later prove to be wrong. To escape the polarizing paralysis, we are convinced that we can - and indeed must - take this kind of approach to reopen our society. Otherwise, we risk consigning millions to economic destitution by trying to live in a zero-risk world that simply does not now, and has never, existed.

## Data availability

No data are associated with this article.
