# Supplementary material for: A conceptual framework for reopening our society during the Covid-19 pandemic
Source: F1000Res. 2020 Jun 8;9:572. [Version 1] doi: 10.12688/f1000research.24352.1 (PMC7885286; doi:10.12688/f1000research.24352.1)
Supplement: Peer review history

# Peer review: Reviewer response for version 1

**DOI:** 10.5256/f1000research.26867.r78042

**Published:** 2021-03-19

**Authors:** Bayarmagnai Weinstein

**Affiliations:** 1 Department of Environmental Health Sciences, School of Public Health, University at Albany, Rensselaer, New York, NY, USA

The authors recommended a framework to help individuals and policymakers to engage in reopening society amidst the COVID-19 pandemic with a benefit-risk balance. At a minimum, this provides an actionable blueprint that can be modified to each user’s context.

 The conceptual basis of the risk index in this article corroborates and warrants the acknowledgment of Blumenshine and Diderichsen’s conceptual framework to delineate the potential causes of disparities in death due to respiratory pandemic as following:

1. likelihood of being exposed to the infectious agent
2. likelihood of contracting the disease, upon exposure to the infectious agent
3. likelihood of receiving timely and effective treatment after disease has developed (Blumenshine et al.,2008 ^1^; Diderichsen et al.,2001 ^2^).

In Gil and Salea’s framework (Figure 1 & 2) the acceptability of risk and perceived value of actions associated with re-opening of selected businesses appear to be modified using remediation measures. I concur with Amrita Rao to clarify the reliability and feasibility of common remediation strategies for direct actions.

 In my view, the recommended framework accounts for the extrinsic factors, such as population density (indoor vs outdoor), type of business (school vs cruise ship), household structure, and age (nursing home and family visits). Even though, prior studies show that vulnerability to COVID-19 has been further defined by intrinsic individual characteristics as following:  

- health risk behaviors (i.e., smoking, excessive alcohol consumption)
- comorbidities (i.e., cardiovascular diseases, obesity, diabetes)
- difficulty to social distancing due to inability to afford unemployment resulting in the reduced exertion of protective measures, healthcare access
- progression of the pandemic over time
- availability and the efficacy of the COVID-19 vaccine

Since the reliability of this one-size-for-all framework would be largely modified by the individual’s intrinsic risk factors as specified above, the authors could have provided or proposed future work to develop user-friendly guidance on how to tailor the framework to individual circumstances.

 Also, for future work, it will be interesting to see if the conceptual model would indicate the data and methods that could bring this to the next level.

Is the topic of the opinion article discussed accurately in the context of the current literature?

Partly

Are arguments sufficiently supported by evidence from the published literature?

Partly

Are all factual statements correct and adequately supported by citations?

Partly

Are the conclusions drawn balanced and justified on the basis of the presented arguments?

Yes

Reviewer Expertise:

Epidemiology and Clinical Research

I confirm that I have read this submission and believe that I have an appropriate level of expertise to confirm that it is of an acceptable scientific standard.

## References

[1] : Pandemic influenza planning in the United States from a health disparities perspective. *Emerg Infect Dis*.2008;14(5) : 10.3201/eid1405.071301 709-15 10.3201/eid1405.071301 18439350PMC2600245

[2] : The Social Basis of Disparities in Health.2001; 10.1093/acprof:oso/9780195137408.003.0002 12-23 10.1093/acprof:oso/9780195137408.003.0002

# Peer review: Reviewer response for version 1

**DOI:** 10.5256/f1000research.26867.r78046

**Published:** 2021-02-15

**Authors:** Amrita Rao

**Affiliations:** 1 Department of Epidemiology, Johns Hopkins Bloomberg School of Public Health, Baltimore, Maryland, USA

In this Opinion Article, the authors argue that much of the public debate around what should and should not be allowed during the COVID-19 pandemic is really an argument about risk tolerance. They note that all decisions made, at the individual and societal level, are a balancing act of how risky an activity might be and how willing we are to absorb that risk. They propose the creation of a risk index that takes into account risk of becoming infected from a certain action, X, and the probability of death or serious harm from participation in that action. Measuring risk in this way, the authors argue, will allow for the *rational* weighing of risk against the social benefits of absorbing that risk. This article is compelling and clearly written.

 From a communication standpoint, I think the use of the two axes and the simplicity of Figure 1 is very useful. It helps us understand the relative risk of different actions in clear terms. It would be helpful, though, to further delineate what the mitigation strategies are and what they might look like.

- Would the mitigation strategies themselves be different in various contexts? For example, mask use at outdoor bar vs. at outdoor national park?
- Can we somehow create a guide for the relative importance of different mitigation strategies (e.g. masks vs. sanitizing)?

To me, I think there are two questions that should play into the risk index: 1) What is the relative risk of different actions? and 2) If I choose to do a certain action, which mitigation strategies should I prioritize?

 The risk index, as I have read it, answers question 1, but I think these gray areas around mitigation strategies is where additional value could be added in terms of helping people make risk-mitigating decisions. The authors argue that this framework at least allows us to start from the same place and have a rational conversation about what is acceptable/debatable/intolerable, but I wanted to see a bit more here.

 The discussion of DALYs was crucial. The authors clearly describe the conceptual similarities between the risk index and the use of DALYs and the advantages of their risk index for different individuals and different scenarios.

Is the topic of the opinion article discussed accurately in the context of the current literature?

Yes

Are arguments sufficiently supported by evidence from the published literature?

Yes

Are all factual statements correct and adequately supported by citations?

Yes

Are the conclusions drawn balanced and justified on the basis of the presented arguments?

Yes

Reviewer Expertise:

Epidemiology; HIV

I confirm that I have read this submission and believe that I have an appropriate level of expertise to confirm that it is of an acceptable scientific standard.

# Peer review: Reviewer response for version 1

**DOI:** 10.5256/f1000research.26867.r64427

**Published:** 2020-07-02

**Authors:** Mauricio T. Caballero

**Affiliations:** 1 Fundación INFANT, Buenos Aires, Argentina; 2 Consejo Nacional de Investigaciones Cientificas y Tecnicas, Buenos Aires, Argentina

A conceptual framework for reopening our society during the Covid-19 pandemic is a well written insight for a new approach to systematically re-opening our society in the midst of the Covid-19 (CV19). Authors propose tools to rationally triage the risk to be compared across different scenarios. The risk should be builded based on two components: 1) the risk of an individual becoming infected due to action ‘X’; and 2) the likely probability of death (or serious harm) if that individual develops CV19. 

 As this is a theorical approach and I considered correct and interesting.

Is the topic of the opinion article discussed accurately in the context of the current literature?

Yes

Are arguments sufficiently supported by evidence from the published literature?

Yes

Are all factual statements correct and adequately supported by citations?

Yes

Are the conclusions drawn balanced and justified on the basis of the presented arguments?

Yes

Reviewer Expertise:

Respiratory viruses disease, pediatrics, epidemiology

I confirm that I have read this submission and believe that I have an appropriate level of expertise to confirm that it is of an acceptable scientific standard.
